# Formation Control for UAVs Considering Safety Constraints Based on Control Barrier Functions with Switched Trajectories and Switching Communication Topologies

**DOI:** 10.3390/s26051477

**Published:** 2026-02-26

**Authors:** Zerui Wei, Xiaoyu Zhang, Yang Song, Rong Guo

**Affiliations:** Beijing Key Laboratory of Robot Bionics and Function Research, Beijing University of Civil Engineering and Architecture, Beijing 100044, China; 2108110023031@stu.bucea.edu.cn (Z.W.); songyang@bucea.edu.cn (Y.S.); guorong@bucea.edu.cn (R.G.)

**Keywords:** UAVs, switched trajectories, switching directed topology, safety, multiple Lyapunov functions

## Abstract

This paper investigates the formation control problem of multi-UAV systems in the presence of switched trajectories and time-varying communication topologies. A distributed formation control protocol is proposed to enable UAVs to track piecewise continuous trajectories while the underlying communication network switches among a finite set of directed graphs. Sufficient and necessary conditions for achieving accurate formation tracking under dual-switching scenarios are derived through stability analysis while the stability of the overall switched system is proven by using multiple Lyapunov functions. To ensure collision avoidance during both trajectory and topology transitions, control barrier functions (CBFs) are employed to construct safety sets, and a quadratic programming(QP)-based optimization framework is designed to modify control inputs in real time. Simulation results demonstrate that the proposed approach effectively coordinates formation tracking, topology switching, and inter-agent safety, offering a solution for UAV collaboration in dynamic and uncertain environments.

## 1. Introduction

Unmanned aerial vehicle (UAV) systems have attracted significant attention in both civilian and military domains due to their versatile applications, including reconnaissance [1], target detection, search and positioning [2], and communication relay networks [3].

Existing approaches to formation control primarily encompass the leader–follower method [4], behaviour-based method [5], and virtual structure method [6]. Although the leader–follower strategy is relatively simple to deploy, it is prone to error propagation within its cascaded framework. This leads to the accumulation of tracking inaccuracies, which can ultimately affect the stability of follower UAVs [7]. Behavior-based techniques, while conceptually intuitive, present difficulties in terms of rigorous stability proof and accurate maintenance of the formation geometry [8]. Virtual structure methods are often inflexible and depend on complex mechanisms to synchronize information across multiple UAVs, reducing their applicability in rapidly changing scenarios [9].

In the last few years, distributed coordination in multi-agent systems (MASs) has attracted extensive research attention, particularly in the context of consensus problems. Based on this background, the application of consensus-based strategies to UAV formation control has become a topic of significant interest. Wei Ren, through his work in [10,11], laid important theoretical and practical foundations in this area. He conducted systematic investigations into consensus algorithms for trajectory tracking and formation coordination, and further validated their feasibility through experiments involving wheeled robots. With ongoing developments in consensus theory, formation control schemes built upon such principles have continued to evolve. For instance, reference [12] established sufficient conditions under which second-order clustered MASs can attain desired formation motions over undirected communication graphs. In [13], the problems of trajectory tracking and formation stabilization were studied for discrete-time MASs, leading to a constructive method for designing consensus-based controllers. Furthermore, the authors of ref. [14] addressed trajectory tracking and formation control in first-order integrator-type MASs operating under switching communication topologies, deriving necessary conditions for achieving stable formation configurations.

In the study of formation-related challenges using consensus methodologies, the team headed by Xiwang Dong has made notable contributions in recent years. The study in reference [15] examines time-varying formation design for high-order linear multi-agent systems (MASs) subject to directed switching communication topologies, adopting a consensus-based strategy. Following this, the authors of references [16,17] address formation control issues via output feedback and state feedback, respectively. In the domain of UAV control, references [18,19] developed consensus-based formation control schemes tailored to directed and undirected switching communication networks, respectively. The algorithms proposed in these works have been effectively tested on UAV platforms, confirming both the feasibility and practical applicability of the adopted approaches.

In recent years, reinforcement learning (RL), as a fundamental artificial intelligence technique, has garnered extensive attention from scholars for its applications in the control domain [20]. Currently, numerous researchers have attempted to apply RL algorithms to multi-agent systems (MASs), giving rise to multi-agent reinforcement learning (MARL) [21]. To address complex cooperative tasks characterized by vast policy spaces and convergence difficulties, hybrid approaches that integrate multiple paradigms have emerged as a prevailing trend, with representative works such as [22]. Reinforcement Learning from Human Feedback (RLHF) represents a critical pathway. In [23], prior knowledge or online assessments from human operators are introduced into the training loop as an additional signal, forming a human–machine collaborative closed loop. This method guides agents to learn safe and compliant policies more rapidly. Inspired by biological swarms in nature, research on emergent coordination mechanisms has also attracted increasing attention [24].

However, real-world deployment of such switching protocols must account for safety constraints. As the density of UAVs increases, ensuring inter-agent collision avoidance during these inherently risky transition phases becomes an important concern. Traditional methods may not adequately address these dual objectives of performance and safety in dynamic and uncertain environments. Safety constraints can effectively improve the efficiency of UAV system safety analysis, especially the control barrier functions (CBFs) proposed in recent years [25,26,27].

The Control Barrier Function (CBF) approach incorporates barrier functions to confine the system state within a safe region, establishing a direct relationship between control inputs and safety constraints. This ensures system stability while complying with all safety requirements [28,29]. Moreover, the method can collect environmental data and employ learning strategies to continuously adapt the safe region. CBF offers a versatile solution for addressing safety concerns in UAV systems. With relatively low computational complexity and strong constraint-handling capability, it is suitable for ensuring operational safety under various conditions, including both known and unknown environments, as well as uncertain system states [30]. It can also be integrated with more aggressive planning algorithms to improve mission efficiency [31]. In recent years, CBF has become an important method for safety control in UAV system research, and has been applied to scenarios such as fixed-area coverage [32], mobile coverage [33], communication maintenance [34], payload transport [35], and aircraft carrier landing [36]. The introduction of safety barrier certificates has further improved the scalability of UAV systems in complex multi-constraint environments [37], broadening the applicability of CBF in UAV operations.

However, there still exist challenges in actual deployment using the safety constraint methods:

1. Ensuring that UAVs form the expected formation, tracking the switched trajectories, while avoiding collisions during the switching process.

2. Considering the switching of trajectory and communication topology at the same time.

The main contributions of this paper are summarized as follows:

1. Unlike works that handle only a single type of switching or plan safety offline, this paper designs a distributed protocol capable of simultaneously handling online switching in both communication topology and reference trajectories, integrated with a real-time safety mechanism based on Control Barrier Functions (CBFs), forming a complete closed-loop solution.

2. By employing the multiple Lyapunov functions approach and deriving a dwell time constraint, this paper provides rigorous stability analysis for UAV swarm systems undergoing simultaneous trajectory and topology switching, proving exponential stability under the proposed conditions.

3. In contrast to many works that apply CBFs in static environments or under fixed topologies, this paper addresses the critical challenge of ensuring collision-free operation throughout the mission by the online construction of safety sets and real-time modification of control inputs via Quadratic Programming (QP) during the highly dynamic process where both trajectories and topology are switching.

The subsequent discussion is structured as follows. Preliminary knowledge and a mathematic model of UAVs are given in Section 2. The control protocol is presented in Section 3. The control barrier functions are proposed in Section 4. Section 5 reports the simulation verification results. Section 6 provides conclusions.

## 2. Problem Formulation

### 2.1. Preliminary Knowledge

Considering a system with *N* UAVs, the interaction topology of the system is characterised as a directed graph $G_{\kappa \left(t\right)}=\left\{Q,\varepsilon_{\kappa \left(t\right)},W_{\kappa \left(t\right)}\right\}$, consisting of a set of nodes $Q=\left\{q_{1},q_{2},\ldots ,q_{N}\right\}$, a set of edges $\varepsilon_{\kappa \left(t\right)}\subseteq \left\{\left(q_{i},q_{j}\right):q_{i},q_{j}\in Q\right\}$ and a weighted adjacency matrix $W_{\kappa \left(t\right)}=\left[w_{ij}^{\kappa \left(t\right)}\right]$. The edges of $G_{\kappa \left(t\right)}$ are denoted by $\varepsilon_{ij}^{\kappa \left(t\right)}=\left(q_{i},q_{j}\right)$. The set of neighbors of node consists of $N_{i}^{\kappa \left(t\right)}=\left\{q_{j}^{\kappa \left(t\right)}\in Q:\left(q_{j},q_{i}\right)\in \varepsilon_{ij}^{\kappa \left(t\right)}\right\}$. The incidence of node is defined as $ind\left(q_{i}^{\kappa \left(t\right)}\right)=\sum_{j=1}^{N}w_{ij}^{\kappa \left(t\right)}$. The incidence degree matrix of $G_{\kappa \left(t\right)}$ is defined as $D_{\kappa \left(t\right)}=\mathrm{diag}\left\{ind\left(q_{i}^{\kappa \left(t\right)}\right),i=1,2,\ldots ,N\right\}$. The Laplacian matrix of $G_{\kappa \left(t\right)}$ is defined as $L_{\kappa \left(t\right)}=D_{\kappa \left(t\right)}-W_{\kappa \left(t\right)}$. $\kappa \left(t\right):\left[0,\infty \right)\to H$ is the switching signal, and $H=\left\{1,2,\ldots ,H\right\}$ is a finite set of topological modes. The existence of a spanning tree for a directed graph is contingent upon the presence of at least one node that possesses directed paths to all other nodes.

**Lemma** **1** 
([38])**.** *It is posited that $L\in R^{N\times N}$ donates the Laplacian matrix of a directed graph G; then,*

*(1) L has one zero eigenvalue at least, and $1_{N}$ is an associated eigenvector, i.e., $L1_{N}=0$.*

*(2) In the event that G possesses a spanning tree, it can be deduced that 0 is a simple eigenvalue of L and that all of the remaining N−1 eigenvalues possess positive real parts.*

**Lemma** **2** 
([39])**.** *For a system $\dot{\varphi }\left(t\right)=M\varphi \left(t\right)$ where M is a 2 × 2 complex matrix and the characteristic polynomial is $f\left(z\right)=z^{2}+a_{1}z+a_{2}$, the system is asymptotically stable if and only if Re(a1)>0 and Re(a1)Re(a1a¯2)−Im(a2)2>0.*

**Lemma** **3** 
([40])**.** *Suppose we have candidate Lyapunov functions $V_{\theta }\equiv V\left(\cdot ,\theta \right)$, and vector fields as in $\dot{x}=f\left(x,\theta \right)\left(\theta \in Z^{+}\right)$ with f(0,θ)=0, for each $\theta \in Z^{+}$. Also, $V:R^{n}\times Z^{+}\to R^{+}$ is continuous. Let S be the set of all switching sequences associated with the system. If for each θ∈S, of all θ, $V_{\theta }$ is Lyapunov-like for $f_{\theta }$ and $x_{\theta }\left(\cdot \right)$, and $V_{\theta }$ satisfies the sequence-nonincreasing condition for $x_{\theta }\left(\cdot \right)$, then the system is stable in the Lyapunov sense.*

### 2.2. Formation Problem

In the context of formation control, the UAV can be expressed as a point-mass system. The dynamics of the UAV system can be modelled as follows:

(1) $$\left\{\begin{array}{cc} {\dot{p}}_{i}\left(t\right) & =v_{i}\left(t\right)\\ {\dot{v}}_{i}\left(t\right) & =u_{i}\left(t\right) \end{array}\right.$$

where $i=1,2,\ldots ,N,p_{i}\left(t\right)\in R^{n}$ denotes the position vector, $v_{i}\left(t\right)\in R^{n}$ denotes the velocity vector, and $u_{i}\left(t\right)\in R^{n}$ denotes the control input of the UAV.

In the following, for simplicity of description, it is assumed that n=1, if not otherwise specified. However, it should be pointed out that similar analysis can also be performed for the higher-dimensional case by using Kronecker product, and all the results hereafter remain valid for n>1.

The UAV system above can be rewritten as follows:

(2) $${\dot{\Gamma }}_{i}\left(t\right)=B_{1}B_{2}^{T}\Gamma_{i}\left(t\right)+B_{2}u_{i}\left(t\right),$$

where $B_{1}={\left[1,0\right]}^{T}$, $B_{2}={\left[0,1\right]}^{T}$, $\Gamma_{i}\left(t\right)={\left[p_{i}\left(t\right),v_{i}\left(t\right)\right]}^{T}$.

Let $F_{i}^{s}\left(t\right)={\left[F_{ip}^{s}\left(t\right),F_{iv}^{s}\left(t\right)\right]}^{T}$ (i=1,2,…,N and $s\in Z^{+}$, s=1,2,…,M indicate which variable will switch) be piecewise continuously differentiable vectors, where $F_{ip}^{s}\left(t\right)$ and $F_{iv}^{s}\left(t\right)$ represent the reference position and velocity components for each UAV, respectively, and $F^{s}\left(t\right)={\left[{\left(F_{1}^{s}\right)}^{T}\left(t\right),{\left(F_{2}^{s}\right)}^{T}\left(t\right),\ldots ,{\left(F_{N}^{s}\right)}^{T}\left(t\right)\right]}^{T}\in R^{2N}$, where

$$F_{i}^{s}\left(t\right)=\left\{\begin{array}{c} {\left[F_{ip}^{1}\left(t\right),F_{iv}^{1}\left(t\right)\right]}^{T},t\in \left[t_{0},t_{1}\right),\\ {\left[F_{ip}^{2}\left(t\right),F_{iv}^{2}\left(t\right)\right]}^{T},t\in \left[t_{1},t_{2}\right),\\ \ldots \ldots \\ {\left[F_{ip}^{M}\left(t\right),F_{iv}^{M}\left(t\right)\right]}^{T},t\in \left[t_{M-1},t_{M}\right), \end{array}\right.$$

and $F_{i}^{s}\left(t\right)$ is continuous in each time interval. Furthermore $F_{i}^{s}\left(t\right)$ satisfies $F_{i}^{s}\left(t_{s}\right)=F_{i}^{s+1}\left(t_{s}\right)$ at the switching instant, where $t_{s}$ is the time for formation switching.

**Definition** **1.** 
*It is asserted that a UAV system is said to realize the formation $F^{s}\left(t\right)$, provided $\lim_{t\to \infty }\left(\Gamma_{i}\left(t\right)-F_{i}^{s}\left(t\right)\right)=0\left(i=1,2,\ldots ,N\right)$ is satisfied.*

Based on the description above and considering the challenges identified in the Introduction, this paper aims to address the following problem: designing a control protocol for system (1) to complete switching formation control and ensure the safety of the formations during switching.

## 3. Control Protocol Design

The formation control protocol is designed as shown below:

(3) $$\begin{array}{cc} u_{i}^{s,\kappa }\left(t\right)= & K_{2}\sum_{j\in N_{i}}w_{ij}^{\kappa \left(t\right)}\left(\left(\Gamma_{j}\left(t\right)-F_{j}^{s}\left(t\right)\right)-\left(\Gamma_{i}\left(t\right)-F_{i}^{s}\left(t\right)\right)\right)\\ \; & +{\dot{F}}_{iv}^{s}\left(t\right)+K_{1}\left(\Gamma_{i}\left(t\right)-F_{i}^{s}\left(t\right)\right), \end{array}$$

where $i=1,2,\ldots ,N,\;K_{1}=\left[k_{11},k_{12}\right]$, $K_{2}=\left[k_{21},k_{22}\right]$ and $k_{11}$, $k_{12}$, $k_{21}$, $k_{22}$ are the constants.

The following discussion first addresses the case where the communication topology is fixed. At this point, formation control protocol is denoted as follows:

(4) $$\begin{array}{cc} u_{i}^{s}\left(t\right)= & K_{2}\sum_{j\in N_{i}}w_{ij}\left(\left(\Gamma_{j}\left(t\right)-F_{j}^{s}\left(t\right)\right)-\left(\Gamma_{i}\left(t\right)-F_{i}^{s}\left(t\right)\right)\right)\\ \; & +{\dot{F}}_{iv}^{s}\left(t\right)+K_{1}\left(\Gamma_{i}\left(t\right)-F_{i}^{s}\left(t\right)\right), \end{array}$$

Let $\Gamma \left(t\right)={\left[\Gamma_{1}^{T}\left(t\right),\Gamma_{2}^{T}\left(t\right),\ldots ,\Gamma_{N}^{T}\left(t\right)\right]}^{T}$, $F_{p}^{s}\left(t\right)={\left[F_{1p}^{s}\left(t\right),F_{2p}^{s}\left(t\right),\ldots ,F_{Np}^{s}\left(t\right)\right]}^{T}$, $F_{v}^{s}\left(t\right)={\left[F_{1v}^{s}\left(t\right),F_{2v}^{s}\left(t\right),\ldots ,F_{Nv}^{s}\left(t\right)\right]}^{T}$. Subsequently, under the condition of employing the formation control protocol (4), the UAV system can be expressed as follows:

(5) $$\begin{array}{cc} \dot{\Gamma }\left(t\right)= & \;\left(I_{N}⊗\left(B_{2}K_{1}+B_{1}B_{2}^{T}\right)-L⊗\left(B_{2}K_{2}\right)\right)\Gamma \left(t\right)\\ \; & -\left(I_{N}⊗\left(B_{2}K_{1}\right)-L⊗\left(B_{2}K_{2}\right)\right)F^{s}\left(t\right)\\ \; & +\left(I_{N}⊗B_{2}\right){\dot{F}}_{v}^{s}\left(t\right). \end{array}$$

Let ${\tilde{\Gamma }}_{i}\left(t\right)=\Gamma_{i}\left(t\right)-F_{i}^{s}\left(t\right)\left(i=1,2,\ldots N\right)$ and $\tilde{\Gamma }\left(t\right)={\left[{\tilde{\Gamma }}_{1}^{T}\left(t\right),{\tilde{\Gamma }}_{2}^{T}\left(t\right),\ldots ,{\tilde{\Gamma }}_{N}^{T}\left(t\right)\right]}^{T}$; UAV system (5) can then be expressed in the following equation:

(6) $$\begin{array}{cc} \dot{\tilde{\Gamma }}\left(t\right)= & I_{N}⊗\left(B_{1}B_{2}^{T}\right)F^{s}\left(t\right)+\left(I_{N}⊗B_{2}\right){\dot{F}}_{v}^{s}\left(t\right)-\dot{F}\left(t\right)\;\\ \; & +\left(I_{N}⊗\left(B_{2}K_{1}+B_{1}B_{2}^{T}\right)\;-\;L⊗\left(B_{2}K_{2}\right)\right)\tilde{\Gamma }\left(t\right). \end{array}$$

Due to the condition of $B_{1}={\left[1,0\right]}^{T}$, $B_{2}={\left[0,1\right]}^{T}$ and ${\dot{F}}^{s}\left(t\right)=\left(I_{N}⊗I_{2}\right){\dot{F}}^{s}\left(t\right)$, UAV system (6) can be rewritten as follows:

(7) $$\begin{array}{cc} \dot{\tilde{\Gamma }}\left(t\right)= & \;\left(I_{N}⊗\left(B_{2}K_{1}+B_{1}B_{2}^{T}\right)-L⊗\left(B_{2}K_{2}\right)\right)\tilde{\Gamma }\left(t\right)\\ \; & +\left(I_{N}⊗B_{1}\right)\left(F_{v}^{s}\left(t\right)-{\dot{F}}_{p}^{s}\left(t\right)\right). \end{array}$$

By defining the tracking error relative to the reference trajectory, the formation tracking problem can be equivalently transformed into a consensus problem of the error system. That is to say, cluster system (5) can achieve formation $F^{s}\left(t\right)$ if and only if cluster system (7) achieves consensus.

Let $\lambda_{i}\left(i=1,2,\ldots N\right)$ be the eigenvalue of the Laplacian matrix *L* of the directed graph *G*, where $\lambda_{1}$ is the eigenvalue corresponding to the eigenvector ${\bar{u}}_{1}=1_{N}$, $\lambda_{2}$ is the eigenvalue corresponding to the eigenvector ${\bar{u}}_{2}$, $\lambda_{3}$ is the eigenvalue corresponding to the eigenvector ${\bar{u}}_{3}$, and so on; 0<Re(λ2)≤⋯≤Re(λN). Let $U^{-1}LU=J$, where $U=\left[{\bar{u}}_{1},{\bar{u}}_{2},\ldots ,{\bar{u}}_{N}\right],U^{-1}={\left[{\tilde{u}}_{1},{\tilde{u}}_{2},\ldots ,{\tilde{u}}_{N}\right]}^{H}$, *J* is the approximate standard form of *L*.

**Theorem** **1.** 
*In the case of fixed communication topology and formation command, UAV system (1) is asymptotically stable under control protocol (4) if and only if the following conditions hold:*

*(a) For any $i\in \left\{1,2,\ldots ,N\right\}$, $\forall j\in N_{i}$, we have*

$\lim_{t\to \infty }\left(\left(F_{iv}^{s}\left(t\right)-F_{jv}^{s}\left(t\right)\right)-\left({\dot{F}}_{ip}^{s}\left(t\right)-{\dot{F}}_{jp}^{s}\left(t\right)\right)\right)=0,$

*(b) For any $i\in \left\{2,3,\ldots ,N\right\}$, we have*

*Re(λi)k22−k12>0,*

*−Im(λi)2k212+(Re(λi)k22−k12)ψi>0,*

*where* 

ψi=(Im(λi)2+Re(λi)2)k21k22+k12k11−Re(λi)(k11k22+k12k21).

**Proof.**  Through Lemma 1, let $J=\mathrm{diag}\left\{0,\bar{J}\right\}$; $\bar{J}$ consists of approximate blocks corresponding to $\lambda_{i}\left(i=2,3,\ldots ,N\right)$. Let $\tilde{U}={\left[{\tilde{u}}_{2},{\tilde{u}}_{3},\ldots ,{\tilde{u}}_{N}\right]}^{H}$, $\varepsilon \left(t\right)=\left({\tilde{u}}_{1}^{H}⊗I_{2}\right)\tilde{\Gamma }\left(t\right)$, $\sigma \left(t\right)=\left(\tilde{U}⊗I_{2}\right)\tilde{\Gamma }\left(t\right)$; then, system (7) can be transformed into the following:

(8) $$\dot{\varepsilon }\left(t\right)=\left(B_{2}K_{1}+B_{1}B_{2}^{T}\right)\varepsilon \left(t\right)+\left({\tilde{u}}_{1}^{H}⊗B_{1}\right)\left(F_{v}^{s}\left(t\right)-{\dot{F}}_{x}^{s}\left(t\right)\right),$$

(9) $$\begin{array}{cc} \dot{\sigma }\left(t\right) & =\left(I_{N-1}⊗\left(B_{2}K_{1}+B_{1}B_{2}^{T}\right)-\bar{J}⊗\left(B_{2}K_{2}\right)\right)\sigma \left(t\right)\\ & \;+\left(\tilde{U}⊗B_{1}\right)\left(F_{v}^{s}\left(t\right)-{\dot{F}}_{p}^{s}\left(t\right)\right). \end{array}$$

It can be inferred from the previous sections that cluster system (5) can realize the formation $F^{s}\left(t\right)$ in each time interval if and only if $\lim_{t\to \infty }\tilde{\Gamma }\left(t\right)=0$. Therefore, according to $\sigma \left(t\right)=\left(\tilde{U}⊗I_{2}\right)\tilde{\Gamma }\left(t\right)$, the following equation can be obtained:

(10) $$\lim_{t\to \infty }\sigma \left(t\right)=0.$$

We first establish that if condition (a) holds, then the following equation holds:

(11) $$\lim_{t\to \infty }(\tilde{U}⊗B_{1})(F_{v}^{s}\left(t\right)-{\dot{F}}_{x}^{s}\left(t\right))=0.$$

If condition (a) holds, then so does the following:

(12) $$\lim_{t\to \infty }\;\left(L⊗B_{1}\right)\;\left(F_{v}^{s}\left(t\right)-{\dot{F}}_{p}^{s}\left(t\right)\right)=0.$$

Substituting $J=U^{-1}LU$ into Equation (12) and left-multiplying both sides simultaneously by $U^{-1}⊗I$, we have the following:

(13) $$\lim_{t\to \infty }\left(\bar{J}\tilde{U}⊗B_{1}\right)\left(F_{v}^{s}\left(t\right)-{\dot{F}}_{x}^{s}\left(t\right)\right)=0.$$

Since *G* has a spanning tree, according to Lemma 1 and the structure of *J*, $\bar{J}$ is nonsingular. Left-multiplying ${\bar{J}}^{-1}⊗I_{2}$ on both sides of (13), i.e., condition (a), creates a sufficient condition for (11).

Let $\tilde{U}=\left[\hat{U},\hat{u}\right]$, where $\hat{U}\in C^{\left(N-1\right)\times \left(N-1\right)}$ and $\hat{u}\in C^{\left(N-1\right)\times 1}$, with $\hat{u}$ being the last column vector of $\tilde{U}$. Since rank $(\hat{U})=N-1$, then from (11),

(14) $$\lim_{t\to \infty }\left(\left[\hat{U},\hat{u}\right]⊗B_{1}\right)\left(F_{v}^{s}\left(t\right)-F_{x}^{s}\left(t\right)\right)=0.$$

Since $U=[{\bar{u}}_{1},{\bar{u}}_{2},\ldots ,{\bar{u}}_{N}]$ is an approximately transformed array, it can be demonstrated that the row vectors are mutually perpendicular, and since $\tilde{U}={\left[{\tilde{u}}_{2},{\tilde{u}}_{3},\ldots ,{\tilde{u}}_{N}\right]}^{H}$ and $u_{1}=1_{N}$, $\tilde{U}1_{N}=0$, $\tilde{U}$ and $1_{N}$ are chunked into the following chunked matrices, respectively:

$$\tilde{U}=\left[\hat{U},\hat{u}\right],1_{N}=\left[\begin{array}{c} 1_{N-1}\\ 1 \end{array}\right],$$

Multiplying the two equations together, we have $\hat{U}1_{N-1}+\hat{u}=0$, i.e.,

(15) $$\hat{u}=-\hat{U}1_{N-1}.$$

Let $\left\{\begin{array}{c} {\bar{F}}_{p}^{s}\left(t\right)={\left[F_{1p}^{s}\left(t\right),F_{2p}^{s}\left(t\right),\ldots ,F_{(N-1)p}^{s}\left(t\right)\right]}^{T}\\ {\bar{F}}_{v}^{s}\left(t\right)={\left[F_{1v}^{s}\left(t\right),F_{2v}^{s}\left(t\right),\ldots ,F_{(N-1)v}^{s}\left(t\right)\right]}^{T}, \end{array}\right.$; then, the following can be obtained from Equations (14) and (15):

(16) $$\begin{array}{cc} \lim_{t\to \infty } & \left(\tilde{U}⊗I_{2}\right)(\left(I_{N-1}⊗B_{1}\right)\left({\bar{F}}_{v}^{s}\left(t\right)-{\dot{\bar{F}}}_{p}^{s}\left(t\right)\right)\\ & -\left(1_{N-1}⊗B_{1}\right)\left(F_{Nv}^{s}\left(t\right)-{\dot{F}}_{Np}^{s}\left(t\right)\right))=0. \end{array}$$

Left-multiplying ${\hat{U}}^{-1}⊗I_{2}$ on both sides of (13), we obtain the following:

(17) $$\lim_{t\to \infty }\left(\left(F_{iv}^{s}\left(t\right)-F_{Nv}^{s}\left(t\right)\right)-\left({\dot{F}}_{ip}^{s}\left(t\right)-{\dot{F}}_{Np}^{s}\left(t\right)\right)\right)=0.$$

It can be demonstrated from Equation (17) that condition (a) is satisfied. Therefore, condition (a) in Theorem 1 is equivalent to Equation (11).

We can find that condition (a) differs from Equation (17) in form. For a detailed proof of equivalence between the two formulas, please refer to Appendix A.

At this point, Equation (9) becomes the following:

(18) $$\dot{\sigma }\left(t\right)\;=\;\left(\;I_{N-1}⊗\left(B_{2}K_{1}\;+\;B_{1}B_{2}^{T}\;\right)\;-\;\bar{J}⊗\left(B_{2}K_{2}\right)\;\right)\sigma \left(t\right).$$

Since $\bar{J}=\mathrm{diag}\left(\lambda_{2},\lambda_{3},\ldots ,\lambda_{N}\right)$,

(19) $$I_{N\;-\;1}\;⊗\left(B_{2}K_{1}\;+\;B_{1}B_{2}^{T}\right)\;-\;\bar{J}⊗\left(B_{2}K_{2}\right)\;=\;\mathrm{diag}\left(\;M_{2},\ldots ,M_{N}\;\right),$$

where

$$\begin{array}{c} M_{i}\;=\;\left(B_{2}K_{1}\;+\;B_{1}B_{2}^{T}\right)-\lambda_{i}B_{2}K_{2}\left(i=2,3,\ldots ,N\right). \end{array}$$

Therefore, the stability of system (18) is equivalent to the stability of the following N−1 subsystems:

(20) $${\dot{\sigma }}_{i}\left(t\right)\;=\;\left(B_{2}\left(K_{1}\;-\;\lambda_{i}K_{2}\right)\;+\;B_{1}B_{2}^{T}\right)\sigma_{i}\left(t\right)\left(i\;=\;2,3,\ldots ,N\right),$$

where

$$B_{2}\left(K_{1}-\lambda_{i}K_{2}\right)+B_{1}B_{2}^{T}=\left[\begin{array}{cc} 0 & 1\\ k_{11}-\lambda_{i}k_{21} & k_{12}-\lambda_{i}k_{22} \end{array}\right].$$

The characteristic polynomial of the subsystem state matrix is given by the following expression: $f_{i}\left(z\right)=z^{2}-\left(k_{12}-\lambda_{i}k_{22}\right)z-\left(k_{11}-\lambda_{i}k_{21}\right)$, where *z* is a complex variable, i=2,3,…,N. As demonstrated in Lemma 2, system (9) is asymptotically stable under condition (b).

Proof ends. □

**Theorem** **2.** 
*In the case of fixed communication topology and switching formation command, if there exist positive definite symmetric matrices $P^{s}$, $Q^{s}$ such that*

(21) $$A^{T}P^{s}+P^{s}A=-Q^{s},$$

*where $A=I_{N}⊗\left(B_{2}K_{1}+B_{1}B_{2}^{T}\right)-L⊗\left(B_{2}K_{2}\right)$, UAV system (1) can achieve stability in the Lyapunov sense under control protocol (4), that is, the UAV system can achieve formation $F^{s}\left(t\right)$.*

**Proof.**  Take the Lyapunov functions in each time interval as

(22) $$V^{s}\left(\tilde{\Gamma }\right)={\tilde{\Gamma }}^{T}P^{s}\tilde{\Gamma },$$

Then, we have the following:

(23) $$\begin{array}{cc} {\dot{V}}^{s} & ={\tilde{\Gamma }}^{T}\left(A^{T}P^{s}+P^{s}A\right)\tilde{\Gamma }\\ \; & =-{\tilde{\Gamma }}^{T}Q^{s}\tilde{\Gamma }\\ \; & \leq -\lambda_{\min}Q^{s}∥\tilde{\Gamma }{∥}^{2}<0. \end{array}$$

Due to $F_{i}^{s}\left(t\right)$ satisfying $F_{i}^{s}\left(t_{s}\right)=F_{i}^{s+1}\left(t_{s}\right)$ at the switching instant and ${\tilde{\Gamma }}_{i}\left(t\right)=\Gamma_{i}\left(t\right)-F_{i}^{s}\left(t\right)$, the error state is continuous at the switching instant, i.e., ${\tilde{\Gamma }\left(t_{s}\right)|}_{s}={\tilde{\Gamma }\left(t_{s}\right)|}_{s+1}$.

For each Lyapunov function of each subsystem, if we take $P^{s+1}<P^{s}$, then

(24) $$V^{s+1}(\tilde{\Gamma }(t_{s+1}))\leq V^{s}(\tilde{\Gamma }(t_{s})).$$

According to (23), (24) and Lemma 3, the UAV system is stable in the Lyapunov sense under the condition that the communication topology is fixed.

The proof ends. □

**Theorem** **3.** 
*In the case of switched communication topology and switching formation command, if there exist positive definite symmetric matrices $P_{h}$, $Q_{h}$ such that*

(25) $$A_{h}^{T}P_{h}+P_{h}A_{h}=-Q_{h}.$$

*where $A_{h}=I_{N}⊗\left(B_{2}K_{1}+B_{1}B_{2}^{T}\right)-L_{h}⊗\left(B_{2}K_{2}\right)$, the dwell time $\tau_{D}$ satisfies $\tau_{D}>\frac{\ln\mu }{2\alpha }$, where $\mu =\frac{\lambda_{\max}\left(P_{h+1}\right)}{\lambda_{\min}\left(P_{h}\right)}$ and α>0. Then, UAV system (1) can achieve exponential stability under control protocol (3), that is, the UAV system can achieve control objectives.*

**Proof.**  Considering system (7), since $A_{h}^{T}P_{h}+P_{h}A_{h}=-Q_{h}$, take the Lyapunov functions for each topology *h* as follows:

(26) $$V_{h}\left(\tilde{\Gamma }\right)={\tilde{\Gamma }}^{T}P_{h}\tilde{\Gamma },$$

For each subsystem under a given topology, we have the following:

(27) $${\dot{V}}_{h}\leq -\lambda_{\min}\left(Q_{h}\right){∥\tilde{\Gamma }∥}^{2}\leq -\frac{\lambda_{\min}\left(Q_{h}\right)}{\lambda_{\max}\left(P_{h}\right)}V_{h}.$$

Take $\alpha_{h}=\frac{\lambda_{\min}\left(Q_{h}\right)}{2\lambda_{\max}\left(P_{h}\right)}$, then

(28) $${\dot{V}}_{h}\leq -2\alpha_{h}V_{h}.$$

Let the switching moment be $t_{h}$, then the following inequality holds:

(29) $$V_{h+1}(\tilde{\Gamma }(t_{h+1}))\leq \mu V_{h}(\tilde{\Gamma }(t_{h})).$$

Considering the time interval $t\in \left[t_{h},t_{h+1}\right)$ and $\kappa \left(t\right)=h$, the system operates under topology *h* within this interval; therefore,

(30) $$V_{h}\left(t\right)\leq e^{-2\alpha \left(t-t_{h}\right)}V_{h}\left(t_{h}\right).$$

where $\alpha =\min_{h}\alpha_{h}$.

**Remark** **1.** *The physical meaning of α is the lower bound of the minimum convergence rate of the overall switched system across all possible communication topologies. It directly determines the minimum dwell time required to guarantee exponential stability: $\tau_{D}>\frac{\ln\;\;\mu }{2\alpha }$. A larger α indicates the stronger intrinsic stability and faster convergence capability of the system, thus allowing for more frequent switching. Conversely, a smaller α requires that switching cannot be too frequent, ensuring the system has sufficient time to converge between switches.*

At the switching moment $t_{h}$,

(31) $$V_{h+1}\left(t_{h}\right)\leq \mu V_{h}\left(t_{h}\right).$$

The following can be obtained by successive recursion:

(32) $$V_{\kappa \left(t\right)}\left(t\right)\leq \mu^{N_{\kappa }\left(0,t\right)}e^{-2\alpha t}V_{\kappa \left(0\right)}\left(0\right),$$

where $N_{\kappa }\left(0,t\right)$ represents the number of switches within [0,t], and $N_{\kappa }\left(0,t\right)\leq \frac{t}{\tau_{D}}$.

Then,

(33) $$V_{\kappa \left(t\right)}\left(t\right)\leq e^{\left(\frac{\ln\mu }{\tau_{D}}-2\alpha \right)t}V_{\kappa \left(0\right)}\left(0\right).$$

When $\tau_{D}>\frac{\ln\mu }{2\alpha }$ is satisfied, the system is exponentially stable.

Proof ends. □

## 4. Safety Constraints

### 4.1. Control Barrier Functions

The Control Barrier Function (CBF) is a method employed to ensure that an agent performs collision-free motion. Constraining the time derivatives of the CBF within prescribed limits ensures the forward invariance of the desired set and guarantees the stability of the system.

Consider the following control system in affine form:

(34) $$\dot{x}=f\left(x\right)+g\left(x\right)u.$$

where $x\in R^{n}$ and $u\in R^{m}$, *f* and *g* are Lipschitz-continuous, and assuming that there exists a set $C\subset R^{n}$, we would like the states of all agents to remain invariant. Therefore, the objective is to design a controller *u* which can guarantee the forward invariance of C, i.e., to make the state of the system (34) remain within C, from the beginning to the end. Assume that the set denoted by C can be defined as the level set of a particular function h(x):

(35) $$C=\left\{x\in R^{n}|h\left(x\right)\geq 0\right\}.$$

**Definition** **2** 
([41])**.** *For a continuously differentiable function $h:R^{n}\to R$ and a given system, system (34), if there exists a local Lipschitz expansion of the class K of functions α and a set $C\subseteq D\subset R^{n}$ such that for all x∈D,*

(36) $$\underset{u\in U}{\sup}\left[L_{f}h\left(x\right)+L_{g}h\left(x\right)u+\alpha \left(h\left(x\right)\right)\right]\geq 0.$$

*then the function h is defined as a Control Barrier Function, defined on D.*

Given a CBF *h*, the feasible set of control inputs is as follows:

(37) $$K\left(x\right)=\{u\in U|L_{f}h\left(x\right)+L_{g}h\left(x\right)u+\alpha \left(h\left(x\right)\right)\geq 0\}.$$

### 4.2. Safety Constraints

As previously outlined, the dual integrator system for UAV can be modified as follows:

(38) $$\begin{array}{cc} {\dot{\Gamma }}_{i}\left(t\right)= & \left(\begin{array}{cc} 0 & I_{n\times n}\\ 0 & 0 \end{array}\right)\;\;\left(\begin{array}{c} p_{i}\left(t\right)\\ v_{i}\left(t\right) \end{array}\right)\;+\;\left(\begin{array}{c} 0\\ I_{n\times n} \end{array}\right)\;u_{i}^{s,\kappa }\left(t\right)\\ = & \;f\left(\Gamma_{i}\right)+g\left(\Gamma_{i}\right)u_{i}^{s,\kappa }. \end{array}$$

Define safety constraints and safety sets, respectively:

(39) $$h_{ij}\left(\Gamma_{i},\Gamma_{j}\right)={∥p_{i}-p_{j}∥}^{2}-{D_{s}}^{2},$$

(40) $$C_{i,j}\;=\;\left\{\left(\Gamma_{i},\Gamma_{j}\right)\;|\;h_{ij}\left(\Gamma_{i},\Gamma_{j}\right)\;=\;{∥p_{i}-p_{j}∥}^{2}\;-\;{D_{s}}^{2}\;\geq 0\;\right\},$$

where $D_{s}$ is the defined safety distance, while $i,j\in \left\{1,2,\ldots ,N\right\}$, and i<j.

To ensure the forward invariance of $C_{i,j}$, the following must be satisfied:

(41) $${\dot{h}}_{ij}+\gamma h_{ij}\geq 0,$$

where γ is the lower bound of the decay rate.

**Remark** **2.** *In the CBF, the decay rate is usually related to the derivative of the function $h\left(x\right)$ and the function value $h\left(x\right)$ itself. Specifically, if there exists a constant γ which makes $\dot{h}\left(x\right)+\gamma h\left(x\right)\geq 0$ hold, then it can be considered a lower bound on the decay rate of the CBF. The size of the lower bound on the decay rate γ affects the strength of the CBF’s “rejection” of the system state. When γ is large, the function h will have a weaker repulsion effect on the unsafe region, and the system state may approach the unsafe region more easily. If γ is small, the function h will have a stronger repulsion effect on the unsafe region, and the system state may approach the unsafe region with more difficulty.*

**Remark** **3.** *(39) depends only on the local position information of agents while the definition and computation are independent of the global communication topology. Regardless of how communication links switch, as long as two agents are within sensing range of each other, the CBFs remain well-defined and can be computed. This provides an absolute safety boundary for the motion of the agents.*

According to (37) and (39), the UAV safety constraints can be constructed as follows:

(42) $$\begin{array}{cc} K_{\mathrm{safe}}=\left\{\left(\Gamma_{i},\Gamma_{j}\right)|\right. & A_{ij}\left(\Gamma_{i},\Gamma_{j}\right){\hat{u}}_{i}\leq b_{ij}\left(\Gamma_{i},\Gamma_{j}\right)\}\\ \; & \;\forall \;i,j\in \{1,2,\ldots ,N\},\;i<j, \end{array}$$

where

$$\begin{array}{c} A_{ij}\left(\Gamma_{i},\Gamma_{j}\right)=-2∥p_{i}-p_{j}∥,b_{ij}\left(\Gamma_{i},\Gamma_{j}\right)=\gamma h_{ij}\left(\Gamma_{i},\Gamma_{j}\right), \end{array}$$

which means that the UAV system is guaranteed to be safe according to Definition 2.

Using QP allows us to achieve the goals of collision avoidance and minimization of changes to the original control inputs:

(43) $$\begin{array}{cc} u_{i}^{*} & =\arg\min_{u}J\left(u\right)=\sum_{i=1}^{N}{∥u_{i}^{s,\kappa }-{\hat{u}}_{i}∥}^{2},\\ s.t.\; & A_{ij}{\hat{u}}_{i}\leq b_{ij},\;\forall i\neq j\\ \; & ∥{\hat{u}}_{i}∥\leq u_{\max},\;\forall i\in \left\{1,2,\ldots ,N\right\}. \end{array}$$

where $u_{i}$ is the original control input and ${\hat{u}}_{i}$ is the input modified by CBFs.

This QP allows the system to operate with high-performance tracking most of the time, and only introduces safety intervention with minimal performance cost when necessary, thereby achieving an optimal balance between the safety and tracking accuracy during switching processes.

## 5. Simulation

**Figure 1:**
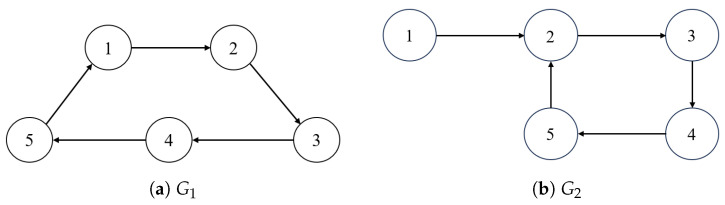
Communication topology $G_{1}$ and $G_{2}$.

**Figure 2:**
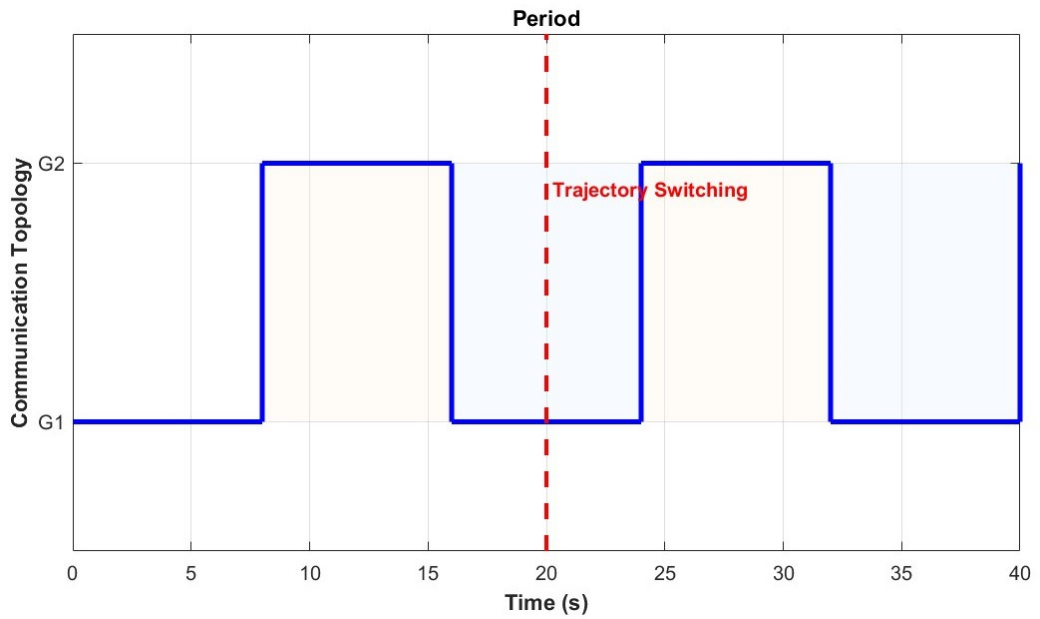
The period of communication topology switching ($\tau_{D}=8$ s).

This section will demonstrate the application of the above method in a simulation environment to prove the validity of the theory proven in Section 3 and Section 4. The communication topology of the UAV system is shown in Figure 1, with a weight of 1 in the communication topology, Figure 2 shows the period of communication topology switching.

### 5.1. Formation Setting

Since the simulation takes place in 3D space, according to the conditions in Theorem 1 and Remark 1, we take $K_{1}=\left[-2,-1.2\right]⊗I_{3},K_{2}=\left[0.35,0.75\right]⊗I_{3}$ and the lower bound of the attenuation rate γ=1.2. Meanwhile, h(x) is designed as (39) and $D_{s}=2$ m.

The initial positions of each UAV are set to (0, 0, 2), (2, 2, 2), (−2, 2, 2), (4, 4, 2), and (−4, 4, 2), respectively, and the five UAVs are numbered in order as *i* (*i* = 1, 2, 3, 4, 5).

In the first stage, i.e., before 20 s, the trajectory of UAV No.1 is formulated as follows:

(44) $$F_{1p}^{s}\left(t\right)=\left[\begin{array}{c} x_{1}\\ y_{1}\\ z_{1} \end{array}\right]=\left\{\begin{array}{c} {\left[\begin{array}{c} t,0,2 \end{array}\right]}^{T},\;t\leq 5,s=1\\ {\left[\begin{array}{c} t,1\;-\;\cos\left(0.5(t\;-\;5)\;\right),\;2 \end{array}\right]}^{T}\;,t\;\geq \;5,s=2 \end{array}\right.$$

where x(t),y(t),z(t) are the components of the UAVs’ reference trajectory in the three coordinate axes. The UAV platform adopted in this simulation is a quadcopter aircraft.

In particular, let the formation trajectory 5 s later be designated c(t), which is the formation centre after formation switching.

The trajectories of UAVs named No.2−5 are as follows:

(45) $$F_{ip}^{s}\left(t\right)={\left[x_{i},y_{i},z_{i}\right]}^{T}=F_{1p}^{s}\left(t\right)+{\left[\Delta x_{i},\Delta y_{i},0\right]}^{T}$$

where *i* = 2, 3, 4, 5, and the variables $\Delta x_{i}$ and $\Delta y_{i}$ represent the initial position deviations of the *i*-th UAV and the first UAV, respectively, in the *x* and *y* axis directions.

At 20 s, the formation is switched, and the subsequent trajectories of the five UAVs are illustrated in the subsequent equation:

(46) $$F_{ip}^{s}\left(t\right)=\left[\begin{array}{c} 4\cos\left(0.5\left(t-20\right)+\frac{2\pi \left(i-1\right)}{5}\right)\\ 4\sin\left(0.5\left(t-20\right)+\frac{2\pi \left(i-1\right)}{5}\right)\\ 2 \end{array}\right]+c\left(t\right),$$

where *i* = 1, 2, 3, 4, 5 and *s* = 3.

### 5.2. Simulation Results

**Figure 3:**
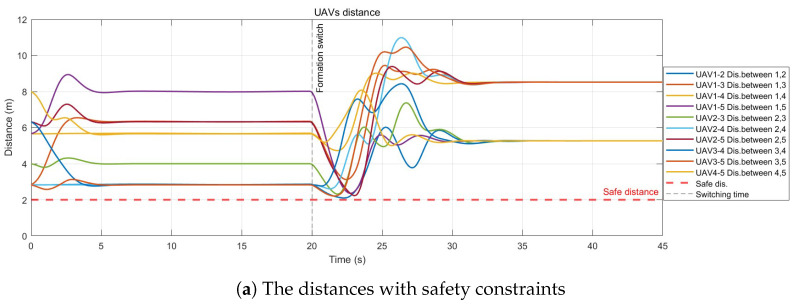

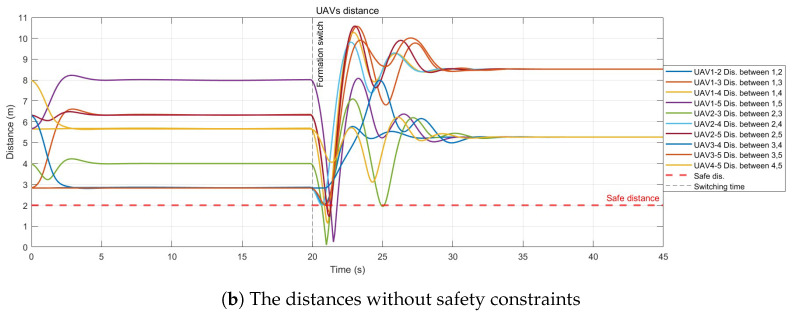
Safety distance monitoring.

Figure 3 shows the distances between each pair of UAVs under conditions with and without safety constraints, respectively. It can be seen that when there exist no safety constraints, collision between UAVs may happen. As shown in Figure 3a, the minimum inter-agent distance throughout the entire mission is 2.03 m, which unmistakenly exceeds the safety threshold $D_{s}=2$ m.

**Figure 4:**
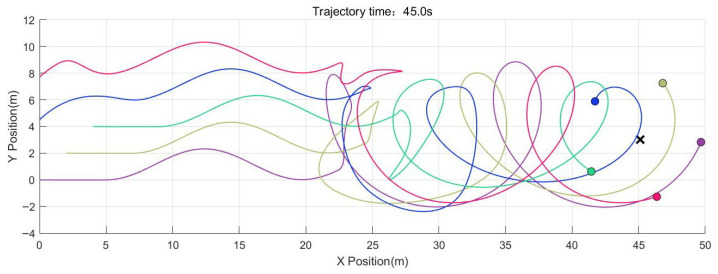
Formation trajectory.

Figure 4 illustrates the formation trajectory under CBFs.

**Figure 5:**
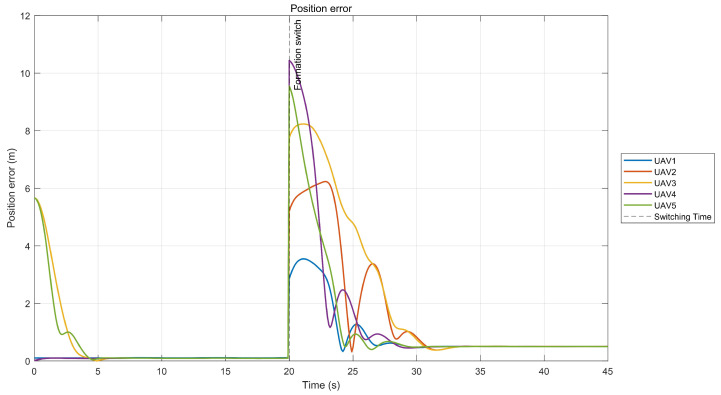
Position error.

Figure 5 illustrates the error between the actual trajectory of the UAVs and the expected trajectory. As shown in Figure 5, the tracking error converges rapidly and remains within 0.15 m in a steady state.

**Figure 6:**
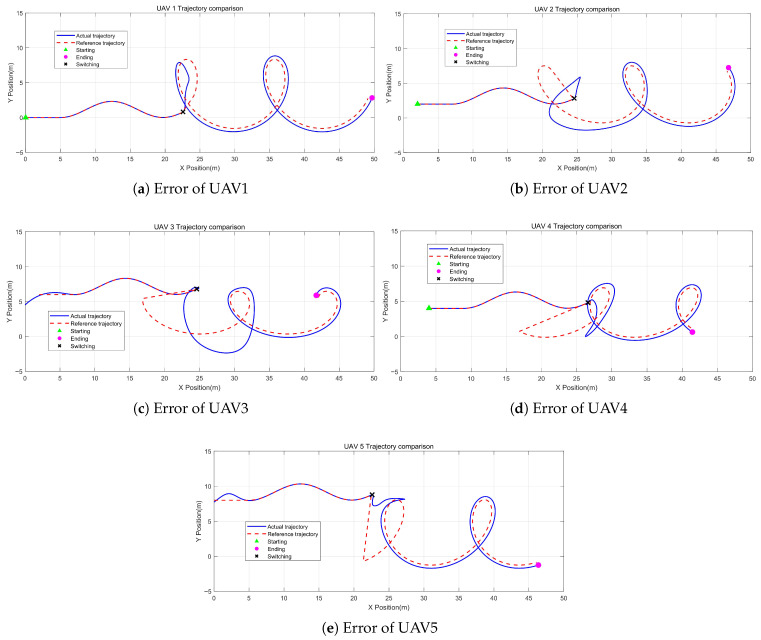
Comparisons.

Figure 6 illustrates the comparison between the actual trajectory and the reference trajectory of each UAV.

It can be seen that there is a certain degree of error between the actual trajectory and the reference trajectory after the formation switching. This result is due to CBFs adjusting the control input to ensure the safety of the formation.

## 6. Conclusions

By addressing communication topology switching and formation trajectory switching, the corresponding novel switching formation control protocol and safety control framework are proposed. Through this control protocol, UAVs can maintain a stable formation structure and perform switched trajectories. Additionally, a proof of the sufficient and necessary conditions for the switching formation control protocol is provided to ensure that each subsystem of the UAV system can be asymptotically stable. Moreover, through the method of multiple Lyapunov functions, it is proven that switching systems can allow us to achieve stability under the condition that each subsystem is asymptotically stable. Furthermore, the CBFs enable the monitoring of distances between UAVs, thereby ensuring safety during trajectory switching. The simulation results validate the effectiveness of the proposed method: all inter-agent distances remain above 2 m throughout the mission, guaranteeing collision-free operation. The tracking error converges to within 0.15 m after each switch, demonstrating high-accuracy formation tracking. Meanwhile, successful formation and topology switching corroborates the dwell time condition for switched-system stability. Future work will focus on extending the proposed method to more realistic dynamics and robust communication protocols, and providing experimental validation with physical UAV platforms.

## Data Availability

Data are contained within the article.

## Appendix A

It is first shown that condition (a) leads to Equation (17).

Assume that condition (a) holds:

(A1) $$\lim_{t\to \infty }\left(\;\left(F_{iv}^{s}\left(t\right)\;-\;F_{jv}^{s}\left(t\right)\right)\;-\;\left({\dot{F}}_{ip}^{s}\left(t\right)\;-\;{\dot{F}}_{jp}^{s}\left(t\right)\right)\right)\;=\;0,j\;\in \;\;N_{i}.$$

Since *G* has a spanning tree, there exists a directed path from any node *i* to the reference node *N*. Noting that the number corresponding to node *i* is $i_{0}$ and the number corresponding to node *N* is $i_{m}$, applying condition (a) to each edge

(A2) $$\left\{\begin{array}{c} \lim_{t\to \infty }\left(\left(F_{i_{0}v}^{s}\left(t\right)-F_{i_{1}v}^{s}\left(t\right)\right)-\left({\dot{F}}_{i_{0}p}^{s}\left(t\right)-{\dot{F}}_{i_{1}p}^{s}\left(t\right)\right)\right)=0,\\ \ldots \\ \lim_{t\to \infty }\left(\left(F_{i_{m-1}v}^{s}\left(t\right)-F_{Nv}^{s}\left(t\right)\right)-\left({\dot{F}}_{i_{m-1}p}^{s}\left(t\right)-{\dot{F}}_{Np}^{s}\left(t\right)\right)\right)=0. \end{array}\right.$$

Adding up all the above equations:

(A3) $$\lim_{t\to \infty }\sum_{k=0}^{m-1}\left(\left(F_{i_{k}v}^{s}\left(t\right)-F_{i_{k+1}v}^{s}\left(t\right)\right)-\left({\dot{F}}_{i_{k}p}^{s}\left(t\right)-{\dot{F}}_{i_{k+1}p}^{s}\left(t\right)\right)\right)=0.$$

Simplifying the above equation yields Equation (17).

Equation (17) is described as for any *i* there are:

$$\lim_{t\to \infty }\left(\left(F_{iv}^{s}\left(t\right)-F_{Nv}^{s}\left(t\right)\right)-\left({\dot{F}}_{ip}^{s}\left(t\right)-{\dot{F}}_{Np}^{s}\left(t\right)\right)\right)=0.$$

Then for $j\in N_{i}$ also satisfies:

$$\lim_{t\to \infty }\left(\left(F_{jv}^{s}\left(t\right)-F_{Nv}^{s}\left(t\right)\right)-\left({\dot{F}}_{jp}^{s}\left(t\right)-{\dot{F}}_{Np}^{s}\left(t\right)\right)\right)=0.$$

Subtracting the above two equations gives condition (a). Therefore, condition (a) and formula (17) are equivalent.
